# Editorial: The importance of faculty development in medical education

**DOI:** 10.3389/fgene.2026.1798159

**Published:** 2026-03-11

**Authors:** Nina Pereza, Goran Hauser, Predrag Jovanović

**Affiliations:** 1 Centre for Improving Teacher Competencies and Communication Skills, Faculty of Medicine, University of Rijeka, Rijeka, Croatia; 2 Department of Medical Biology and Genetics, Faculty of Medicine, University of Rijeka, Rijeka, Croatia; 3 Department of Internal Medicine, Faculty of Medicine, University of Rijeka, Rijeka, Croatia; 4 Department of Internal Medicine, Clinical Hospital Centre Rijeka, Rijeka, Croatia; 5 Clinic for Internal Diseases, University Clinical Center Tuzla, Tuzla, Bosnia and Herzegovina; 6 Faculty of Medicine, University of Tuzla, Tuzla, Bosnia and Herzegovina

**Keywords:** faculty development, healthcare profession, medical education, medical teacher, teaching innovation

## 1 Faculty development: a global imperative for excellence and innovation in higher education

Medical education is built on a paradox that has been recognized for decades: the people who carry the main responsibility for teaching future healthcare professionals are rarely trained as educators. Physicians and other healthcare professionals are prepared extensively for clinical and research roles, yet their educational role is often assumed to be intuitive, learned informally, or acquired on the job. At the same time, the quality of any healthcare system is tightly linked to the quality of medical education, and therefore to the competence, motivation, and continued professional growth of medical teachers.

Consequently, in recent years, faculty development has emerged as a cornerstone of high-quality medical education, yet the availability of structured, sustained opportunities for professional educational development of medical teachers remains uneven across the world. As editors of this thematic Research Topic, based in Croatia and Bosnia and Herzegovina, we are acutely aware that in most of Europe faculty development is still fragmented, informal, or entirely absent. Teaching excellence is still not widely regarded as something developed through intentional professional preparation. In contrast, at the University of Rijeka, Faculty of Medicine in Croatia, we have been in the process of building a faculty development system from the ground up since 2017, driven by a growing recognition that effective teaching is neither solely intuitive nor incidental, but a professional competence requiring support, time, and institutional commitment (Pereza et al.).

This lived experience of both absence and emergence shaped our decision to curate this Research Topic, which brings together diverse perspectives on faculty development as a catalyst for personal growth, educational quality, and institutional advancement. Our aim was to bring together work that examines how medical and healthcare professions institutions prepare and support their teaching staff, how faculty development is organized and evaluated, and how these efforts intersect with curriculum reform, assessment, technology, and global health priorities. We were particularly interested in contributions from diverse settings, including those where formal faculty development structures are still emerging.

This Research Topic assembles 30 articles and 163 authors from Europe, Asia, Africa, Australia, and the Americas, reflecting a truly global perspective on medical teacher professional development. The contributions span multiple healthcare professions, including medicine, nursing, pharmacy, and laboratory sciences, and cover a range of educational contexts from undergraduate curricula to postgraduate and clinical training. We have classified the articles into four major Research Topic of interest, including faculty development strategies, global lessons from the local medical education systems, education in specific medical domains, teaching and learning models emerging from evidence-based innovation, and healthcare profession students of the future.

The collected articles reinforce that faculty development, when intentional and evidence-based, can systematically improve educator competencies, foster innovation, and enhance student outcomes across regions, demonstrating that structured academic support is a global priority rather than a local privilege. As such, faculty development extends beyond simple enhancement of teacher competencies to a strategic initiative advancing individual and institutional growth and recognition.

While research spans diverse contexts, the Research Topic resonates strongly with our own experience in Croatia and Bosnia and Herzegovina, where opportunities for structured faculty development remain limited. By bringing together this international evidence, we aim to inform the creation of robust faculty development systems in regions like ours, highlighting the potential for personal growth, institutional advancement, and improved educational outcomes. The international contributions demonstrate that faculty development is adaptable to local contexts while maintaining universal principles, offering practical guidance for regions with emerging educational infrastructures.

## 2 Faculty development strategies: individual and institutional transformation

Effective faculty development is increasingly recognized as a strategic lever for both individual educator growth and institutional transformation. Several contributions in this Research Topic demonstrate that faculty development is most effective when it is conceived not as a series of disconnected workshops, but as a strategic institutional designation anchored in dedicated structures and informed by systematic needs assessment.

Two articles in this Research Topic illustrate that competency-based approaches improve educator satisfaction, retention, and institutional performance, underscoring the value of aligning professional development with organizational goals and measurable outcomes. Competency-based frameworks, as exemplified by initiatives at Johns Hopkins School of Medicine (Robertson et al.), provide structured guidance across domains including teaching, leadership, scholarship, and system-level contributions. Aligning faculty development programs with defined competencies not only supports professional identity formation but also legitimizes educational work within promotion and recognition pathways, fostering sustained institutional impact. Key lessons from global implementations emphasize stakeholder engagement, flexibility in programming, and the visibility of educator contributions as essential elements for successful adoption.

Local initiatives, such as the Modern and Practical Medical Education faculty development program at the University of Rijeka, Faculty of Medicine in Croatia, illustrate the value of a data-informed, context-specific approach (Pereza et al.). Through a three-phase mixed method study, including 360-degree needs assessment, competency mapping, and outcome evaluation, medical teachers demonstrated highly significant gains across thirty teacher competencies, while institutional curricula were enhanced through targeted educational projects in partnership with students. Such programs underscore that comprehensive and sustainable faculty development must be both evidence-based and responsive to the unique needs of the institution and its learners. These findings emphasize that tailoring faculty development to actual institutional needs generates measurable improvements at both the individual and systemic levels, transforming educational quality and practice, and becoming a matter of institutional prestige in the long-term.

## 3 Global lessons from the local medical education systems

The global contributions in this Research Topic reveal the diversity of medical education systems, their specificities and challenges, as well as the critical role of faculty development in addressing systemic disparities. In Sub-Saharan Africa, collaborations between Moi University in Kenya and Brown University in the United States strengthened nephrology education by targeted interventions, increasing clinician confidence from 9% to over 96% within 4 years (Owino et al.). Similarly, cross-national analyses in Brazil, China, and Latin America highlight the interplay of institutional resources, regional disparities, mentorship structures, and curricular integration in shaping educational outcomes (Rufino et al., Liu et al., Li et al., Menezes et al.).

These studies demonstrate that faculty development cannot be isolated from system-level considerations. Structural inequities, workload, and limited resources directly impact the capacity of educators to implement innovative teaching models. For instance, continuing medical education programs in Sichuan Province, China, revealed that hospital characteristics, program duration, and governmental support influenced completion rates, emphasizing the need for strategic allocation of resources alongside pedagogical training (Liu et al.). Furthermore, analyses of Latin America’s physician-scientist mentorship highlight that systemic barriers, such as funding limitations, fragmented curricula, and insufficient mentorship—must be addressed to cultivate a research-capable workforce (Menezes et al.). Together, these studies underscore that faculty development programs must be integrated into broader systemic strategies to ensure sustainable, high-quality medical education, and that effective faculty development required close collaboration and synchronization between policymakers and experts in medical education.

## 4 Education in specific medical domains: from population health to genetics

Faculty development extends beyond foundational teaching skills to specialized competencies across medical domains. The articles in this Research Topic emphasize once again that every medical field is specific and requires equally specific faculty development programs and teaching strategies for the mastery of learning outcomes in the field.

In primary care, developing a population health mindset among general practitioners involves training in epidemiology, data analysis, and community engagement (Jerjes et al.). Competency in this area requires reflective practice, longitudinal exposure to patient populations, and the ability to translate systemic insights into practical interventions. Global evidence demonstrates that structured population health training equips GPs to address health inequities and improves outcomes at the community level, highlighting the need for faculty capable of guiding these complex learning experiences.

Similarly, discipline-specific initiatives in neurology (Fan et al.), anaesthesiology (Neskovic et al.), laboratory medicine (Yang et al.), hematology (Xie et al.), genetics (Charles et al.), pharmacy (Gharib et al.), and nursing illustrate (Huang et al.) the breadth of educational challenges and innovations. Programs integrating computer-based simulations, laboratory-result-oriented modular teaching, evidence-based leadership training, and genetic diagnostics in Africa demonstrate that faculty development must encompass both technical and pedagogical expertise. Importantly, these interventions improve not only educator competence but also learner outcomes, critical thinking, and patient-centered care. The DDD-Africa genetics training, for instance, demonstrates how faculty development programs can create regional networks of trained professionals, expanding access to specialized care and fostering research capacity in under-resourced contexts.

## 5 Teaching and learning models: evidence-based innovation

Instructional strategies underpinning effective medical education are a central theme in this Research Topic, reflecting the growing recognition that how we teach is as important as what we teach. In addition, an important lesson learned is that no innovation can be introduced in education without first investing in teacher education.

Evidence from organ system-based curricula in China demonstrates that localized, clinically integrated teaching enhances short-term clinical skills such as history-taking and physical examination, even if long-term licensing exam outcomes remain comparable, emphasizing the value of context-sensitive curriculum design (Zhang et al.). Similarly, the implementation of the BOPPPS model—both alone and combined with situational teaching in intensive care training—has shown significant improvements in residents’ theoretical knowledge, clinical reasoning, critical thinking, and teamwork skills, demonstrating that structured, active learning approaches can transform the learning environment (Liu et al.; Liu et al.). Network meta-analyses of nursing education highlight that peer-assisted learning approaches, including “Peer Sister Teaching,” outperform traditional methods in fostering humanistic care competencies, reflecting the power of collaborative, learner-centered strategies (Meixia et al.).

Objective Structured Clinical Examinations (OSCEs) further illustrate the critical role of evidence-based evaluation, providing reliable, standardized assessments of practical competencies while revealing areas for curricular improvement (Yang et al.). Innovations such as laboratory-result-oriented modular teaching for hematology interns or simulation-based training in pharmacy show that integrating hands-on, problem-focused approaches improves operational skills, clinical reasoning, and learner satisfaction. Moreover, these approaches demonstrate the importance of aligning teaching methods with competency frameworks, learner readiness, and real-world clinical tasks, ensuring that educational interventions translate into meaningful practice improvements and authentic medical education. Collectively, these studies underscore that evidence-based teaching and learning strategies, spanning active learning, simulation, peer collaboration, and structured assessment, enhance knowledge acquisition, clinical competence, professional attitudes, and critical thinking. The consistent, measurable impact across disciplines reinforces the necessity of investing in innovative pedagogies to prepare learners for complex, interdisciplinary healthcare environments.

## 6 HPE students of the future: preparing adaptive, ethical, and technologically literate professionals

The future of medical education depends on shaping learners who are not only clinically competent but also socially responsible, ethically grounded, and digitally literate (Miguez-Pinto et al.). Articles in this Research Topic examine health behaviors, engagement, and professional identity among students, revealing gaps in adherence to health-promoting behaviors, the impact of teachers’ appreciation on academic performance, and the mediating role of engagement in managing stress in English-medium instruction programs (Ip et al., Fatima et al., Saadati et al., Liu et al., Masutha et al., Ruck et al., Xiang et al., Al Zahrani et al., Hailu et al.).

Innovations in curricular design, such as interventions addressing weight stigma, integration of artificial intelligence, and global public health education, highlight the importance of preparing students for a complex healthcare landscape. Nursing students’ perceptions of their future careers, with an emphasis on altruism, ethics, and personal growth, reflect broader trends in workforce motivation and the need for supportive educational and professional environments. A multicenter survey explores faculty perspectives on artificial intelligence adoption, identifying enthusiasm for its potential but also concerns about ethics, equity, and the risk of overreliance on automated tools (Al Zahrani et al.). The authors argue that faculty development must help educators critically appraise artificial intelligence applications, understand their limitations, and integrate them into curricula in ways that support rather than replace human judgement and interaction.

Collectively, these studies reinforce that faculty development and student preparation are inextricably linked: skilled, engaged, and supported educators are essential to cultivating the next-generation of adaptable, empathetic, and innovation-driven health professionals. Evidence also shows that fostering student engagement, appreciation, and structured mentorship directly improves learning outcomes, stress adaptation, and professional identity formation, emphasizing that student success is a reflection of faculty quality and institutional commitment.

## 7 Looking ahead: recommendations and future directions

The strong engagement with this Research Topic, reflected in its 163 authors and more than 76,000 views, suggests that faculty development is no longer a peripheral concern but a central pillar of health professions education. The collective evidence and discussion highlight five priorities for advancing faculty development in medical education:

1. Local Ownership Over Universal Models: There is no single ideal approach to faculty development. International collaboration can inform practice, but sustainable programs must be locally developed and adapted to institutional and healthcare contexts, informed by data from students, educators, and patients.
2. Coordinated and Visible Strategies: National support and centralized institutional faculty development units are essential to sustaining growth by coordinating strategy, mentorship, evaluation, and recognition across institutions.
3. Institutional and Administrative Commitment: Long-term faculty development requires explicit administrative buy-in, including protected time, adequate resources, and alignment with promotion and recognition systems.
4. No Teaching Innovation without Teacher Education: Educational innovation cannot be successfully introduced or sustained without first investing in teacher education. Faculty must be prepared to understand, implement, and critically engage with new pedagogies, technologies, and curricular reforms for innovation to translate into meaningful learning and improved care.
5. But Beyond Improving Teacher Competencies: Improving teacher competencies is only the starting point. Effective faculty development must also cultivate leadership, advocacy, research capacity, digital literacy, and evolving clinical and educational roles.

In conclusion, faculty development is not merely a professional luxury, it is a strategic imperative that shapes the quality of medical education, the capabilities of the health workforce, and ultimately, the care of patients. This Research Topic demonstrates that, through intentional, evidence-based, and context-sensitive initiatives, faculty development can serve as a catalyst for personal growth, institutional advancement, and the preparation of health professionals for a rapidly changing global landscape. By highlighting global innovations, identifying gaps, and offering actionable recommendations, we hope to inspire educators, policymakers, and institutions to embrace faculty development as central to the mission of medical education. The evidence clearly shows that structured, innovative, and contextually relevant faculty development programs improve educator competence, learner outcomes, and healthcare delivery worldwide, establishing faculty development as a cornerstone of sustainable, high-quality medical education.

